# Supplementary material for: Effects of Low Benzoic Acid Concentrations on Growth and Substrate Utilization in Black Soldier Fly Larvae
Source: Insects. 2025 Nov 12;16(11):1155. doi: 10.3390/insects16111155 (PMC12653391; doi:10.3390/insects16111155)
Supplement: Supplementary file 1 [file insects-16-01155-s001.zip › insects-3965369-supplementary.pdf]

Supplementary Materials

Effects of low benzoic acid concentrations on growth and substrate utilization in black soldier fly larvae

Thor Brødsted Christiansen and Niels Thomas Eriksen

Department of Chemistry and Bioscience, Aalborg University, Fredrik Bajers Vej 7H,
DK-9220 Aalborg, Denmark

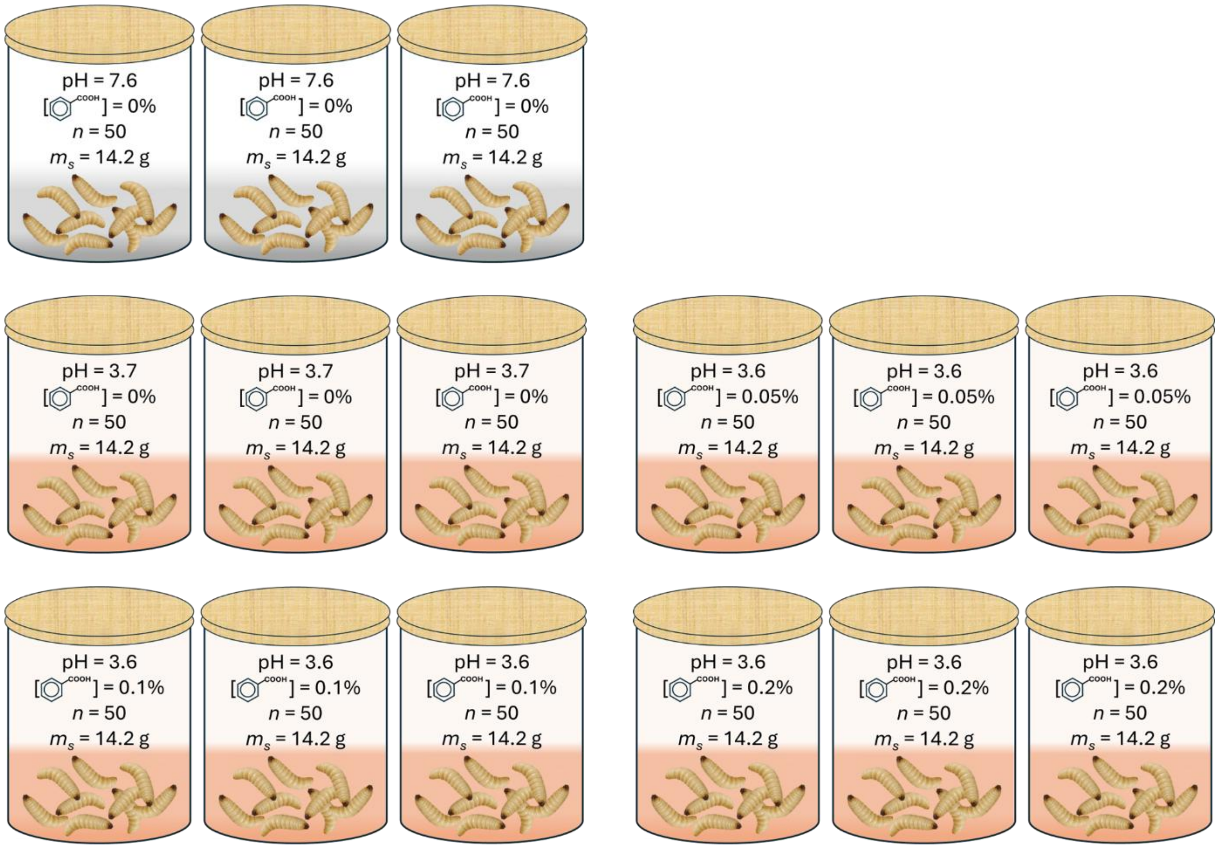

Figure S1. Experimental design for assaying the effect of low concentrations of benzoic acid at low pH. Larval performances were investigated in triplicate at 0-0.2% benzoic acid and initial substrate pH 3.6-3.7 (symbolised by reddish containers). Larvae reared at 0% benzoic acid and initial substrate pH 7.6 were included as control (symbolised by greyish containers).

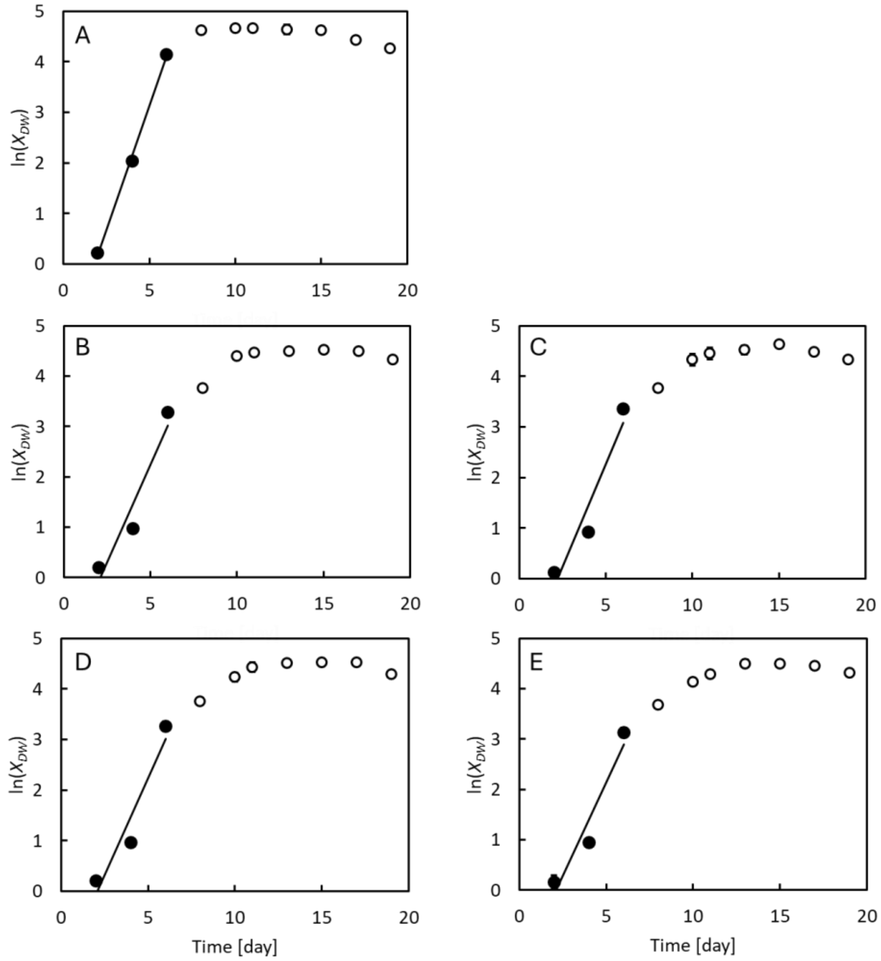

Figure S2. Logarithmic transformed dry weights of BSF larvae reared at different concentrations of benzoic acid at low pH. Solid symbols indicate the exponential growth phase. A. Control, 0 % benzoic acid and initial substrate pH 7.6. B-E. Larvae reared at 0, 0.05%, 0.1%, or 0.2% benzoic acid, respectively, and initial substrate pH 3.6. Specific growth rates estimated from the slope of the regression lines are listed in Table S1. Data points indicate average values ± standard deviation of 3 replicate cultures. Original data in Fig. 1.

Tabel S1. Experimental conditions, variables, and parameters from BSF larvae reared at different concentrations of benzoic acid at low pH (Figs. 1-4 and S1-S2). The experiment was started Day 2 (age of starter larvae) and terminated Day 19.

[Benzoic acid]		Control	0%	0.05%	0.1%	0.2%
Initial experimental conditions						
pH_2		7.6	3.7	3.6	3.6	3.6
$m_{substrate,2}$	g DM	14.2	14.2	14.2	14.2	14.2
n_2		50	50	50	50	50
*X_2	mg DW	1.2 ± 0.1	1.2 ± 0.1	1.1 ± 0.1	1.2 ± 0.1	1.2 ± 0.2
$m_{larvae,2}$	g DM	0.06 ± 0.0	0.06 ± 0.00	0.06 ± 0.00	0.06 ± 0.00	0.06 ± 0.01
$m_{substrate,2}$:$n_{larvae,2}$	mg	284	284	284	284	284
Experimental results						
pH_{19}		7.9	4.6	4.8	4.8	4.7
$m_{frass,19}$	g DM	3.48 ± 0.1	3.48 ± 0.0	4.08 ± 0.2	4.38 ± 0.1	4.48 ± 0.1
n_{19}		49 ± 1	50 ± 1	48 ± 3	50 ± 1	50 ± 0
Survival rate		0.99 ± 0.01	0.99 ± 0.01	0.96 ± 0.05	0.99 ± 0.01	1.00 ± 0.00
$m_{larvae,19}$	g DM	3.54 ± 0.06	3.81 ± 0.06	3.66 ± 0.06	3.66 ± 0.04	3.75 ± 0.04
μ	day^{-1}	0.98 ± 0.02	0.77 ± 0.04	0.81 ± 0.03	0.76 ± 0.02	0.74 ± 0.02
Model parameters						
X_{max}	mg DW	106 ± 4	95 ± 1	102 ± 5	96 ± 5	89 ± 1
μ_{max}	day^{-1}	1.21 ± 0.01	0.76 ± 0.01	0.70 ± 0.05	0.71 ± 0.01	0.67 ± 0.04
Performance indicators						
NGE_{avg}		0.55 ± 0.01	0.44 ± 0.01	0.49 ± 0.03	0.50 ± 0.02	0.49 ± 0.02
$NGE_{avg,DW}$		0.49 ± 0.01	0.39 ± 0.01	0.44 ± 0.03	0.44 ± 0.02	0.44 ± 0.01
SRR		0.76 ± 0.00	0.76 ± 0.00	0.72 ± 0.01	0.70 ± 0.01	0.69 ± 0.01
SCE		0.32 ± 0.01	0.35 ± 0.00	0.35 ± 0.01	0.36 ± 0.01	0.38 ± 0.00

*The dry weight of starter larvae was estimated from wet weight measurements assuming 30% DW content

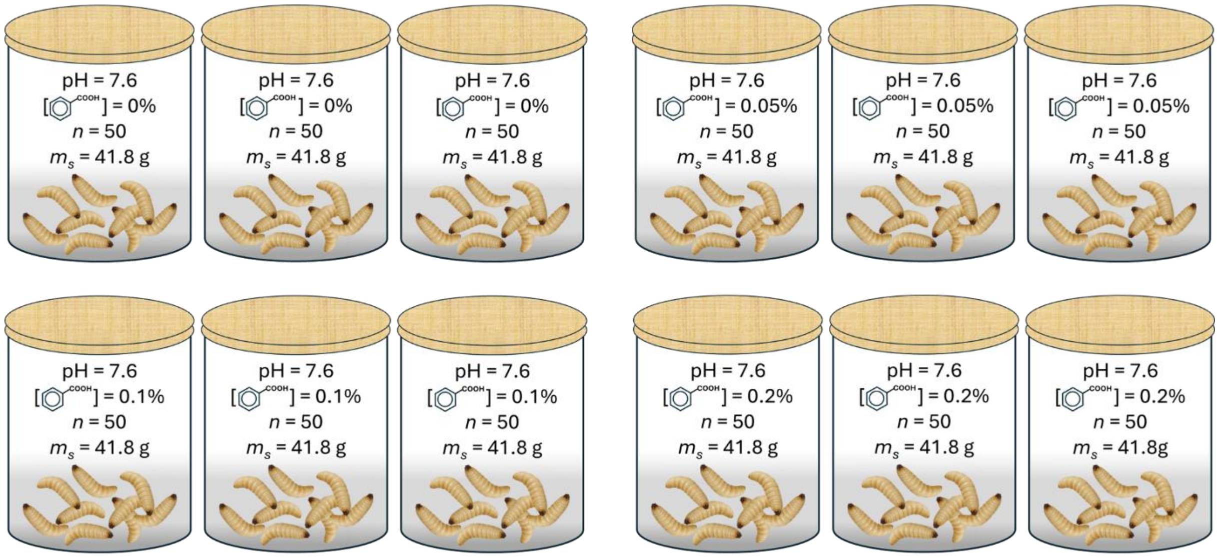

Figure S3. Experimental design for assaying the effect of low concentrations of benzoic acid at neutral pH. Larval performances were investigated in triplicate at 0-0.2% benzoic acid and initial substrate pH 7.6 (symbolised by greyish containers).

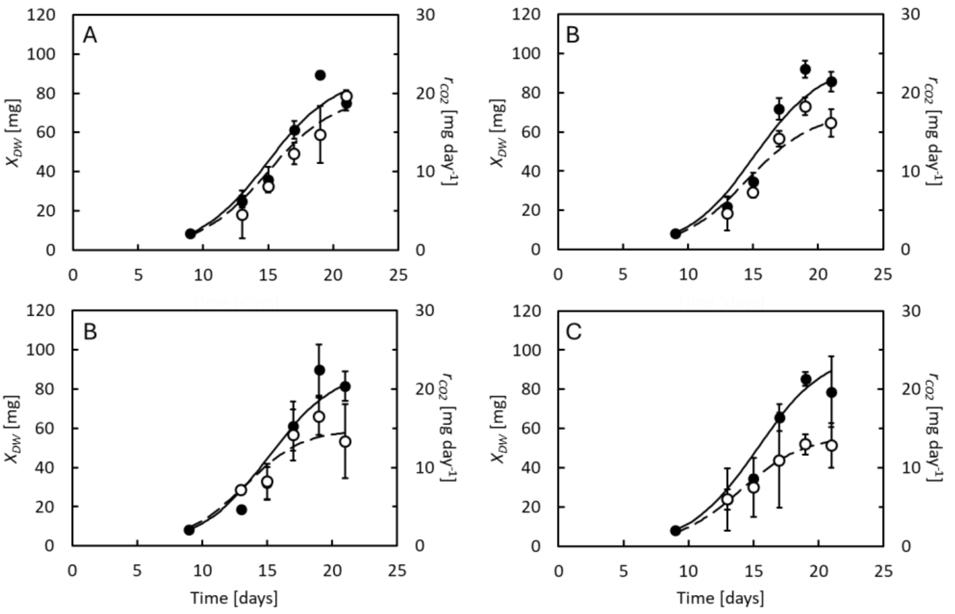

Figure S4. Dry weight (●) and CO_2 production rate (○) of BSF larvae reared at different concentrations of benzoic acid at neutral pH. A-D. Larvae reared at 0, 0.05%, 0.1%, or 0.2% benzoic acid, respectively. Data points indicate average values ± standard deviation of 3 replicate cultures. Curves are modelled by Eqs. 1, 4 and 6.

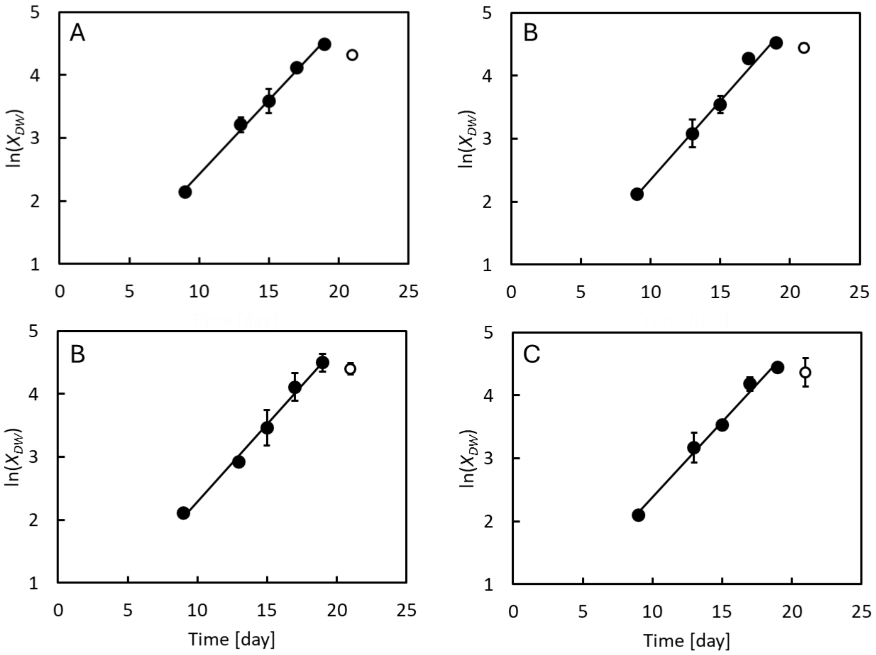

Figure S5. Logarithmic transformed dry weights of BSF larvae reared at different concentrations of benzoic acid at neutral pH. Solid symbols indicate the exponential growth phase. A-D. Larvae reared at 0, 0.05%, 0.1%, or 0.2% benzoic acid, respectively. Specific growth rates estimated from the slope of the regression lines are listed in Table S2. Data points indicate average values ± standard deviation of 3 replicate cultures. Original data in Fig. S4.

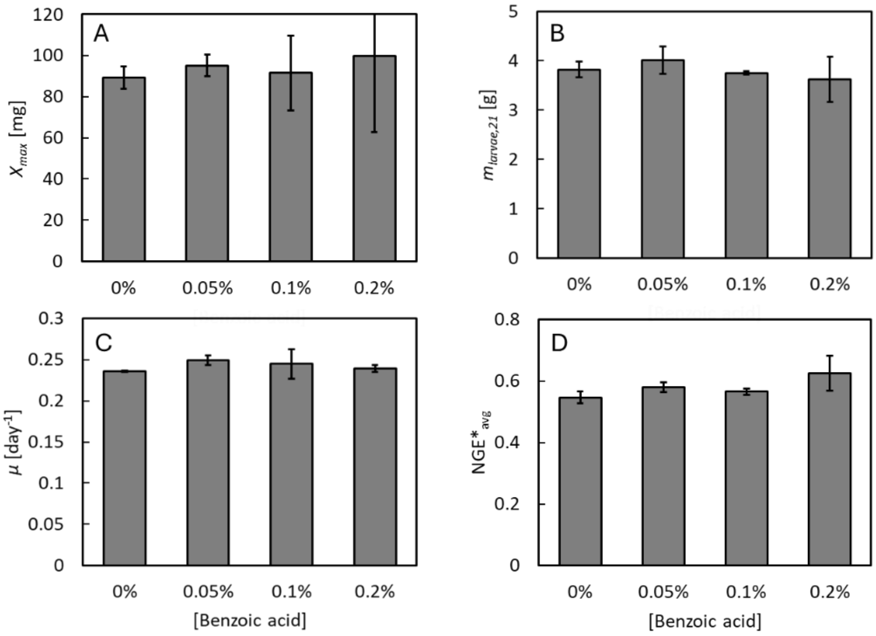

Figure S6. Performance of BSF larvae reared different concentrations of benzoic acid and neutral pH. A. Maximal DW, X_{max}. B. Total harvested larval dry matter on Day 21. C. Specific growth rate during exponential growth phase, μ. D. Average carbon net growth efficiency, NGE*$_{avg}$. Bars indicate average values ± standard deviation of 3 replicate cultures.

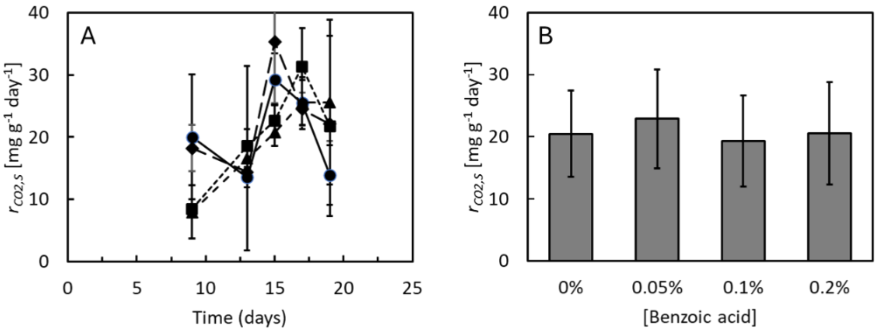

Figure S7. CO_2 production rates from feed substrates at neutral pH. A. Daily CO_2 production rates from feed substrates, $r_{CO2,S}$ sampled from cultures of BSF larvae and no benzoic acid (●), 0.05% benzoic acid (♦), 0.1% benzoic acid (▲), and 0.2% benzoic acid (■). Data points indicate average values ± standard deviation of 3 replicate cultures. B. Average CO_2 production rates from feed substrates at neutral pH and different concentrations of benzoic acid (black bars). Bars indicate average values ± standard deviation of 3 replicate cultures.

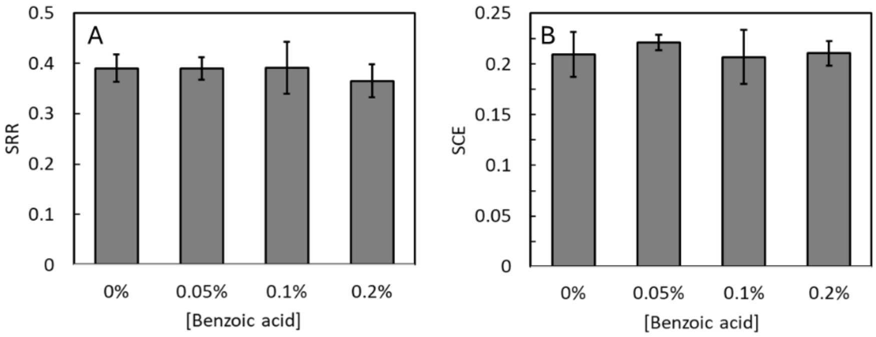

Figure S8. Substrate reduction rates and substrate conversion efficiencies at neutral pH. A. Substrate reduction rate (SSR). B. Substrate conversion efficiency (SCE). Bars indicate average values ± standard deviation of 3 replicate cultures.

Tabel S2. Experimental conditions, variables, and parameters from BSF larvae reared at different concentrations of benzoic acid at neutral pH (Figs. S3-S8). The experiment was started Day 9 (age of starter larvae) and terminated Day 21.

[Benzoic acid]		0%	0,05%	0,1%	0,2%
Initial experimental conditions					
pH		7.6	7.6	7.6	7.6
$m_{substrate,9}$	g DM	41.8	41.8	41.8	41.8
n_9		50	50	50	50
$*X_9$	mg DW	8.5 ± 0.1	8.4 ± 0.2	8.2 ± 0.2	8.2 ± 0.2
$m_{larvae,9}$	g DM	0.43 ± 0.00	0.42 ± 0.01	0.41 ± 0.01	0.41 ± 0.01
$m_{substrate,9}$:$n_{larvae,9}$	mg	836	836	836	836
Experimental results					
$m_{frass,21}$	g DW	25± 1	25 ± 1	25 ± 2	27 ± 1
n_{21}		48 ± 1	47 ± 4	49 ± 1	47 ± 3
Survival rate		0.96 ± 0.02	0.95 ± 0.08	0.97 ± 0.01	0.93 ± 0.05
$m_{larvae,21}$	g DM	3.8 ± 0.2	4.0 ± 0.3	3.8 ± 0.0	3.6 ± 0.5
μ	day^{-1}	0.24 ± 0.00	0.25 ± 0.01	0.24 ± 0.02	0.24 ± 0.00
Model parameters					
X_{max}	mg DW	85 ± 5	96 ± 5	103 ± 18	99 ± 37
μ_{max}	day^{-1}	0.37 ± 0.01	0.37 ± 0.01	0.34 ± 0.05	0.35 ± 0.05
Performance indicators					
NGE_{avg}		0.56 ± 0.02	0.56 ± 0.01	0.57 ± 0.01	0.59 ± 0.06
$NGE_{avg,DW}$		0.50 ± 0.02	0.50 ± 0.01	0.51 ± 0.01	0.53 ± 0.05
SRR		0.39 ± 0.03	0.39 ± 0.02	0.39 ± 0.05	0.36 ± 0.03
SCE		0.21 ± 0.02	0.22 ± 0.01	0.21 ± 0.03	0.21 ± 0.01

*The dry weight of starter larvae was estimated from wet weight measurements assuming 30% DW content

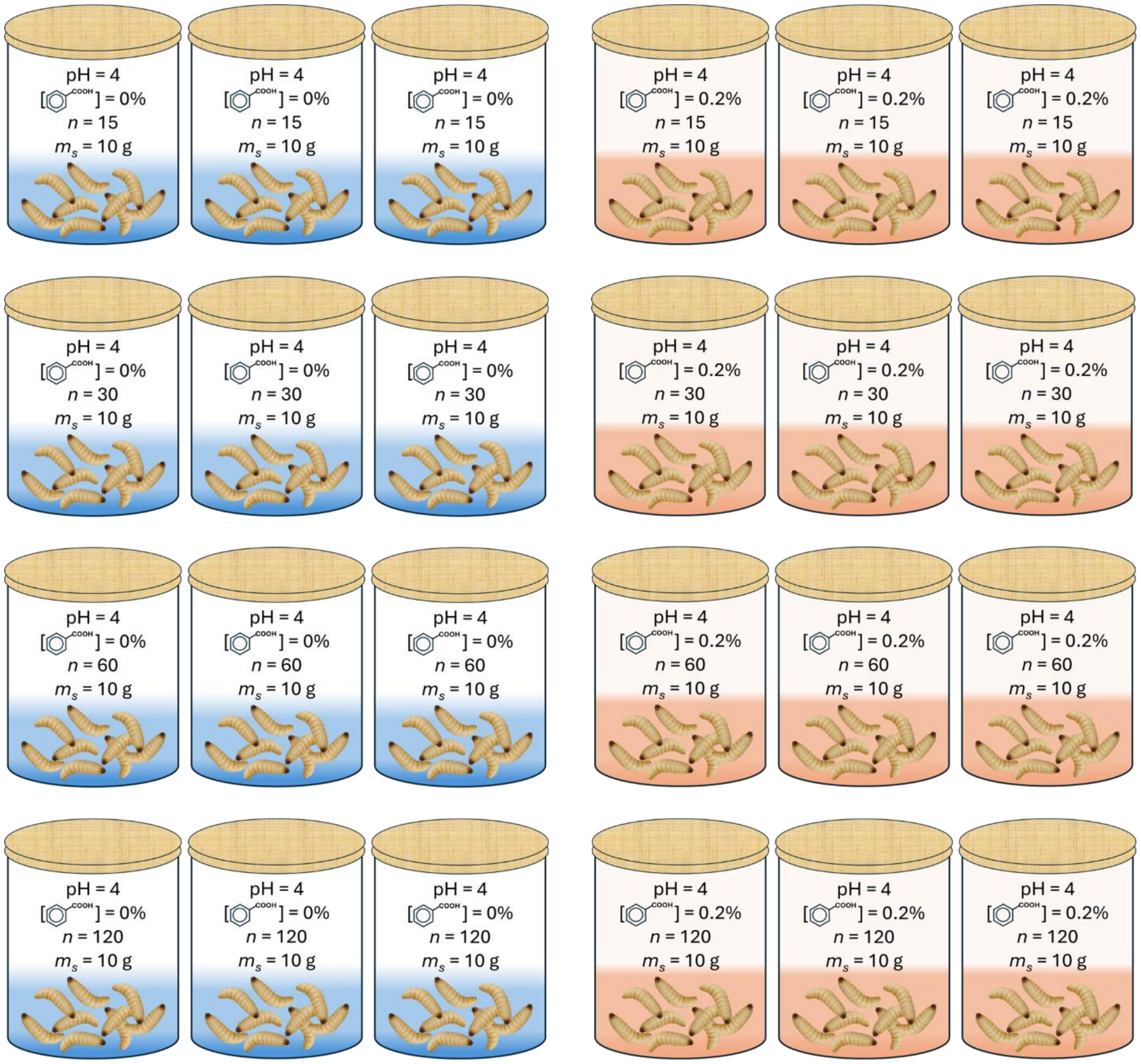

Figure S9. Experimental design for assaying the effect of low concentrations of benzoic acid at low pH and variable larval densities. Larval performances were investigated in triplicate at 0 (symbolised by blueish containers) or 0.2% benzoic acid and initial substrate pH 4 (symbolised by reddish containers).

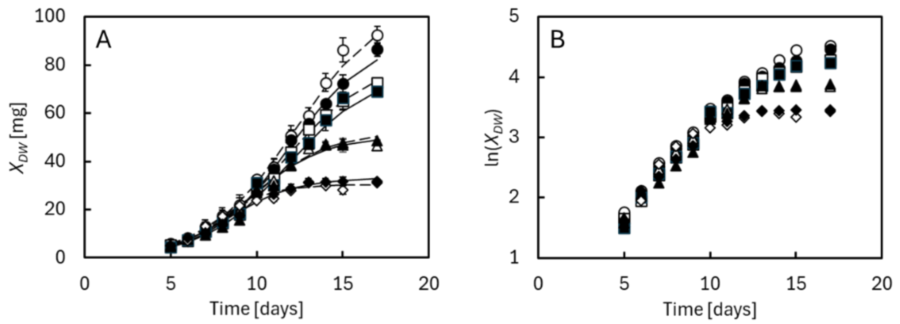

Figure S10. Dry weight of BSF larvae reared at low pH and different larval densities with and without benzoic acid. A. Dry weight of BSF larvae reared at larval densities of 15 (○, ●), 30 (□,■),60 (▲, △), or 120 (♦, ♢) larvae per 8.4 g of chicken feed, initial feed substrate pH 4, and no benzoic acid (open symbols) or 0.02% benzoic acid (solid symbols). Symbols indicate average values ± standard deviation of 3 replicate cultures. B. Ln-transformed dry weight of BSF larvae. Same symbols as in A. Data points indicate average values ± standard deviation of 3 replicate cultures. Data from the exponential growth phase, Day 5-10 used for estimation of specific growth rates (Table S3).

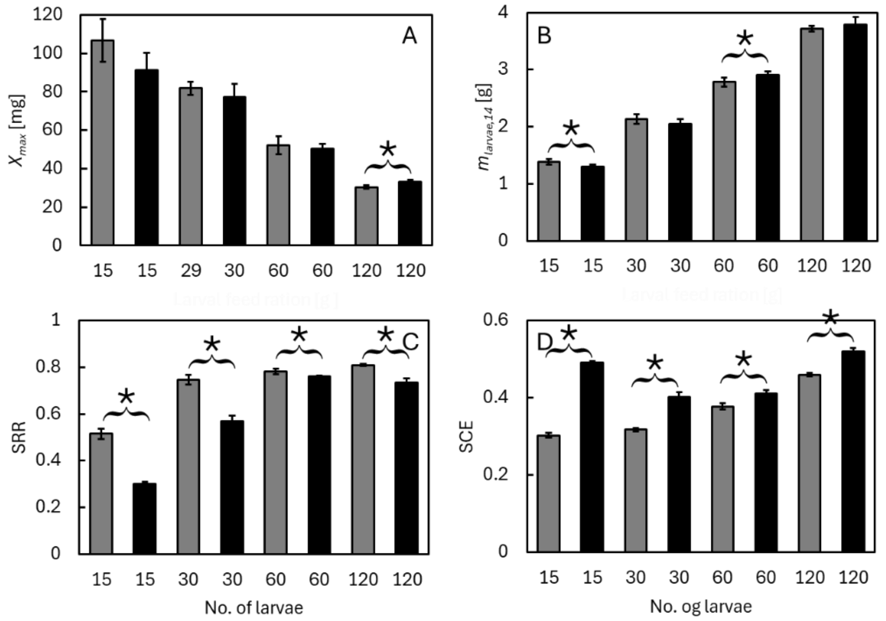

Figure S11. Performance of BSF larvae reared at low pH and different larval densities with and without benzoic acid. BSF larvae reared without benzoic acid (grey bars) or 0.2% benzoic acid (black bars). A. Maximal DW, X_{max}. B. Total larval dry matter at end of experiment. C. Substrate reduction rate, SSR. D. Substrate conversion efficiency, SCE. Bars indicate average values ± standard deviation of 3 replicate cultures. Stars indicate significant differences (t-test, $p < 0.05$) between trials at the same larval density with and without benzoic acid. Growth curves are shown in Fig. S10.

Tabel S3. Experimental conditions, variables, and parameters from BSF larvae reared at low pH and different larval densities with and without benzoic acid (Figs. 5 and S10-S11). The experiment was started Day 5 (age of starter larvae) and terminated Day 14.

No. of BSF larvae		15	15	30	30	60	60	120	120
[Benzoic acid]	%	0	0.2	0	0.2	0	0.2	0	0.2
Initial experimental conditions									
pH_5		4	4	4	4	4	4	4	4
$m_{substrate,5}$	g DM	8.4	8.4	8.4	8.4	8.4	8.4	8.4	8.4
n_5		15	15	30	30	60	60	120	120
*X_5	mg DW	5.8 ± 0.3	4.6 ± 0.6	5.2 ± 0.2	4.7 ± 0.4	5.3 ± 0.4	4.9 ± 0.3	5.1 ± 0.2	5.0 ± 0.2
$m_{larvae,5}$	g DM	0.09 ± 0.01	0.07 ± 0.01	0.16 ± 0.01	0.13 ± 0.01	0.32 ± 0.03	0.31 ± 0.02	0.61 ± 0.03	0.60 ± 0.02
$m_{substrate,5}$:$n_{larvae,5}$	mg	557	557	278	278	139	139	70	70
Experimental results									
$m_{frass,14}$	g DM	4.0 ± 0.2	5.8 ± 0.1	2.1 ± 0.2	3.6 ± 0.2	1.8 ± 0.1	2.0 ± 0.0	1.6 ± 0.0	2.2 ± 0.1
n_{14}		15 ± 0	15 ± 0	29 ± 1	30 ± 1	60 ± 1	60 ± 1	120 ± 1	120 ± 1
Survival rate		1.00 ± 0.00	1.00 ± 0.00	0.98 ± 0.02	0.99 ± 0.02	0.99 ± 0.01	1.00 ± 0.02	1.00 ± 0.00	1.00 ± 0.01
$m_{larvae,14}$	g DM	1.39 ± 0.05	1.30 ± 0.04	2.13 ± 0.08	2.05 ± 0.09	2.78 ± 0.08	2.91 ± 0.06	3.72 ± 0.04	3.78 ± 0.14
μ	day^{-1}	0.34 ± 0.02	0.37 ± 0.03	0.33 ± 0.02	0.36 ± 0.01	0.32 ± 0.02	0.31 ± 0.01	0.32 ± 0.01	0.31 ± 0.02
Model parameters									
X_{max}	mg DW	107 ± 11	91 ± 9	82 ± 3	77 ± 7	52 ± 5	50 ± 2	30 ± 1	33 ± 1
μ_{max}	day^{-1}	0.39 ± 0.03	0.43 ± 0.04	0.40 ± 0.01	0.41 ± 0.02	0.46 ± 0.05	0.48 ± 0.01	0.62 ± 0.05	0.51 ± 0.03
Performance indicators									
SRR		0.52 ± 0.02	0.30 ± 0.01	0.74 ± 0.02	0.57 ± 0.02	0.78 ± 0.01	0.76 ± 0.00	0.81 ± 0.00	0.74 ± 0.02
SCE		0.30 ± 0.01	0.49 ± 0.00	0.32 ± 0.00	0.40 ± 0.01	0.38 ± 0.01	0.41 ± 0.01	0.46 ± 0.00	0.52 ± 0.01

*The dry weight of starter larvae was estimated from wet weight measurements assuming 30% DW content
